# Distal Consequences of Mucosal Infections in Intestinal and Lung Inflammation

**DOI:** 10.3389/fimmu.2022.877533

**Published:** 2022-04-28

**Authors:** Felipe Melo-González, Javiera Sepúlveda-Alfaro, Bárbara M. Schultz, Isidora D. Suazo, David L. Boone, Alexis M. Kalergis, Susan M. Bueno

**Affiliations:** ^1^ Millennium Institute on Immunology and Immunotherapy, Pontificia Universidad Católica de Chile, Santiago, Chile; ^2^ Departamento de Genética Molecular y Microbiología, Facultad de Ciencias Biológicas, Pontificia Universidad Católica de Chile, Santiago, Chile; ^3^ Departamento de Ciencias Biológicas, Facultad de Ciencias de la Vida, Universidad Andrés Bello, Santiago, Chile; ^4^ Department of Microbiology and Immunology, Indiana University School of Medicine-South Bend, South Bend, IN, United States; ^5^ Departamento de Endocrinología, Facultad de Medicina, Pontificia Universidad Católica de Chile, Santiago, Chile

**Keywords:** gut-lung axis, infectious diseases, inflammation, microbiota, metabolites

## Abstract

Infectious diseases are one of the leading causes of morbidity and mortality worldwide, affecting high-risk populations such as children and the elderly. Pathogens usually activate local immune responses at the site of infection, resulting in both protective and inflammatory responses, which may lead to local changes in the microbiota, metabolites, and the cytokine environment. Although some pathogens can disseminate and cause systemic disease, increasing evidence suggests that local infections can affect tissues not directly invaded. In particular, diseases occurring at distal mucosal barriers such as the lung and the intestine seem to be linked, as shown by epidemiological studies in humans. These mucosal barriers have bidirectional interactions based mainly on multiple signals derived from the microbiota, which has been termed as the gut-lung axis. However, the effects observed in such distal places are still incompletely understood. Most of the current research focuses on the systemic impact of changes in microbiota and bacterial metabolites during infection, which could further modulate immune responses at distal tissue sites. Here, we describe how the gut microbiota and associated metabolites play key roles in maintaining local homeostasis and preventing enteric infection by direct and indirect mechanisms. Subsequently, we discuss recent murine and human studies linking infectious diseases with changes occurring at distal mucosal barriers, with particular emphasis on bacterial and viral infections affecting the lung and the gastrointestinal tract. Further, we discuss the potential mechanisms by which pathogens may cause such effects, promoting either protection or susceptibility to secondary infection.

## Introduction

Infectious diseases occur when a pathogen infects a primary tissue and can cause both local and systemic damage in the host. Mucosal tissues such as the lung and the intestine are particularly susceptible to infectious microorganisms, which may overcome local innate immune responses and cause inflammation and tissue damage. Lung and intestinal tissue are composed of several layers of protective molecules and cells that prevent mucosal damage. Specialized epithelial cells called goblet cells secrete mucins (Muc2 in the gut and Muc5b and Muc5ac in the lung), which are complex glycoproteins that prevent direct contact with potentially harmful pathogens in both mucosal barriers forming a mucus barrier (1). The epithelial cell layer secretes antimicrobial molecules such as defensins in both tissues, directly affecting pathogen survival. Immune cells are crucial sentinels of the intestine and lung, with several types of innate immune cells continuously sampling environmental antigens and able to initiate inflammatory responses such as dendritic cells and macrophages [reviewed in (2–4)]. Following infection, inflammatory signals can recruit other innate and adaptive inflammatory immune cells into the site of infection. The migration of immune cells into mucosal tissues is dependent on the expression of homing factors, which recognize particular chemokines and adhesion molecules. In particular, the chemokine receptors CCR7, GPR15, CCR9, CCR10 and the integrin α4β7 have been identified as gut homing receptors, whereas CCR1, CCR2, CCR3, CCR4, CCR5, CXCR3, and CXCR4 have been reported as lung homing receptors (5, 6).

Increasing evidence suggests that lung disease can be linked to gastrointestinal illness as shown by epidemiological studies in humans, which indicate a strong correlation between inflammatory chronic lung diseases such as asthma and chronic obstructive pulmonary disease (COPD) and gastrointestinal diseases, including IBD and irritable bowel disease (7). In addition, both mucosal barriers can establish a bidirectional interaction based on multiple-signals mainly influenced by the microbiota, which has been termed as the gut-lung axis (8). Although enteric or pulmonary pathogens usually do not colonize both mucosal barriers, there is increasing evidence showing distal consequences of infectious diseases that may be attributed to either altered immune responses in the primary site of infection or changes in the normal microbiota inhabiting mucosal tissues, especially the intestine. Trillions of microorganisms inhabit and/or transit through the intestinal tract, and the crosstalk between the intestinal microbiota and intestinal cells is key to maintain barrier integrity and prevent mucosal damage (9). The beneficial role of the microbiota has been mainly explained by their relevant role in breaking down the dietary fiber into short-chain fatty acids (SCFA) and controlling the abundance of other molecules, such as amino acids and bile acids, which can play differential roles in the modulation of immune cells (10). Whole metagenomic sequencing and metabolic analysis have recently linked the human microbiota with the 95% of fecal metabolite content and 34% of blood metabolites, highlighting the importance of the microbiota in generating metabolites that can play immunomodulatory roles (11). Importantly, these metabolites can enter the circulation system and modulate immune responses in distal tissues. Indeed, diets high in fiber or fermented food induce changes in the metabolites and markers of inflammation in circulation. In particular, a high fermented food diet causes increased microbiota diversity together with decreased inflammatory signals in circulation (12). An extensive amount of work has revealed the important role of the intestinal microbiota and microbiota-derived signals in maintaining intestinal homeostasis and generating protection against enteric pathogens. This review will first describe the local effects of the gut microbiota and associated metabolites and then will discuss how infectious diseases in mucosal barriers can affect immune responses in distal tissues.

## The Role of the Microbiota and Gut Metabolites in Gut Homeostasis and Infection

The intestinal microbiota composition is crucial in limiting the infection with enteric pathogens, as shown in murine models of intestinal infection such as *Salmonella enterica* ser. Typhimurium (*S.* Typhimurium) and *Citrobacter rodentium*. These beneficial effects of the microbiota are partly attributed to the competition for metabolites and the production of bactericidal molecules by the commensal microbiota, as has been highlighted in studies with germ-free mice, who cannot eradicate these pathogens (13–16). *S.* Typhimurium can successfully outcompete the commensal bacteria by overcoming antimicrobial responses and exploiting the host immune response to suppress the growth of intestinal microbiota (17, 18). Consistent with this, resistance to *S.* Typhimurium colonization seems to be determined by the commensal intestinal microbiota (19–22). This protective effect is dependent on the induction of anti-bacterial responses mediated by IFN-γ and IL-17, which are produced by both CD3^-^ innate lymphocytes and T cells (19). These cytokines are needed to prevent bacterial dissemination, which correlates with disease severity (19). Moreover, colonization resistance to *C. rodentium* seems to be determined by commensals with similar metabolic requirements for monosaccharides (14). Thus, commensal bacteria can control colonization of enteric bacteria by the regulation of immune responses but also by regulating metabolite availability.

Commensal bacteria can prevent the colonization of other enteric pathogens such as *Vibrio cholerae* and *Clostridioides difficile*, the latter a pathogen responsible for high morbidity and mortality in hospitalized patients. For example, the presence of *Bacteroides vulgatus*, which is highly abundant in healthy mice and humans, can suppress *V. cholerae* infection in germ-free mice (23). Similarly, fecal microbiota transplant from healthy individuals improves survival and recovery in patients infected with *C. difficile*, confirming the protective role of the microbiota against infection in humans (24–26). In addition, mice harboring microbiota of ulcerative colitis patients exhibit increased susceptibility to *C. difficile*, which is prevented by the administration of microbiota from healthy humans and the commensal bacterium *Phascolarctobacterium*. This microbe reduces succinate availability and prevents *C. difficile* colonization (27). Interestingly, IL-22-mediated mucus glycosylation favors the growth of these commensal bacteria, a pathway that is altered in ulcerative colitis patients, which explains their increased susceptibility to *C. difficile* infection (27). Similarly, colonization of germ-free mice with dysbiotic microbiota from patients with diarrhea showed increased susceptibility to *C. difficile* infection by increasing abundance in amino acids such as proline, which may favor *C. difficile* growth (28). Therefore, alterations in the commensal microbiota composition may drive increased susceptibility to enteric infection.

**Figure 1:**
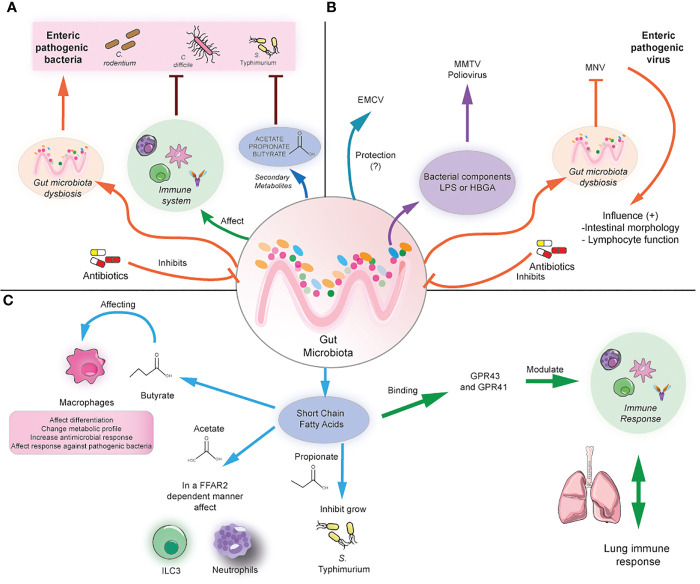
Modulation of local and distal infection by the gut microbiota. **(A)** The gut microbiota can prevent enteric infection by modulating the immune cell responses and/or the production of secondary metabolites such as SCFA. In contrast, antibiotics induce gut microbiota dysbiosis, causing susceptibility to bacterial enteric infection. **(B)** The gut microbiota can affect the infection with enteric viruses differently, depending on the virus. For example, it could play a protective role in the case of ECMV, but it may promote the infection with MMTV and poliovirus by the binding with bacterial components and subsequent facilitation of viral infection. In antibiotic-treated mice, MNV can compensate for the beneficial effects of the gut microbiota by promoting anti-inflammatory responses. **(C)** The gut microbiota can produce SCFA, which in turn can modulate macrophages directly, ILC3, and neutrophil function by binding through the GRP41/43 receptors. In addition, SCFA can directly inhibit bacterial growth. In addition, SCFA can distally modulate lung immune responses.

Studies in animal models using antibiotic treatments have shown the importance of the microbiota and derived metabolites in resistance against enteric pathogens. Some antibiotics such as clindamycin, cefoperazone, and ampicillin render increased susceptibility to *C. difficile* (29–31). Cefoperazone induces profound changes in the metabolome, reducing secondary bile acids, glucose, free fatty acids, and dipeptides while increasing the abundance of the primary bile acid taurocholate and sugars such as mannitol, fructose, sorbitol, raffinose, and stachyose. This metabolic environment favors *C. difficile* spore germination and growth (30). Furthermore, administration of non-pathogenic *Clostridium scindens* increases infection resistance to *C. difficile* in murine models due to its ability to synthesize secondary bile acids, which inhibit *C. difficile* growth (**Figure 1A**) (31). Clindamycin also renders susceptibility to *Vibrio cholerae* infection by altering the gut microbiota and metabolome, reducing SCFA (23). Thus, antibiotics can play a detrimental role by impacting the microbiota and metabolome composition, increasing the susceptibility to various pathogenic microbes.

## The Role of Commensal Microbiota in Enteric Viral Infection

In contrast to the widely beneficial effects of the commensal microbiota against bacterial enteric infection, several studies have reported that commensal bacteria within the mammalian intestinal tract can enhance enteric virus infections through a variety of mechanisms. For example, mouse mammary tumor virus (MMTV) infects the intestine and then is transmitted to the host offspring, but germ-free mice cannot transmit the virus, highlighting the role of the microbiota in this process. Indeed, MMTV binds to bacterial lipopolysaccharide (LPS) and then suppresses antiviral immune responses through TLR-4 signaling and subsequent IL-10 expression, inducing tolerance to the virus (32). Similarly, infection with poliovirus, a virus transmitted through the fecal-oral route, in antibiotic-treated mice renders them less susceptible to disease, as viral replication is enhanced by binding to LPS (33). In addition, it has been demonstrated that LPS promotes poliovirus virion stability and increases binding to the poliovirus receptor (34). In line with this, murine norovirus (MNV), responsible for causing gastroenteritis, can infect B cells *in vitro* and *in vivo* models in the presence of histo-blood group antigen (HBGA)-expressing enteric bacteria, and its replication is reduced *in vivo* when the intestinal microbiota was depleted (35). Antibiotic-treated mice are also less susceptible to murine norovirus, and the virus persistence is decreased in an IFN-λ-dependent manner (36). A similar phenotype was reported for the infection with reovirus, which was less severe in antibiotic-treated mice. However, it has not been shown that LPS or other bacterial components are responsible for this effect (33). Thus, commensal bacteria may facilitate binding to host receptors, modulation of immune responses, and viral replication.

Some recent studies highlight that the microbiota may play protective roles against viral infection. For example, in the case of encephalomyocarditis virus (EMCV), a virus that infects the intestine that spreads systemically and affects the nervous system, the depletion of the microbiota increases neuropathogenesis, viremia, and viral burden in brains and mortality due to EMCV infection. This protective effect is mediated by the commensal *Blautia coccoides*, which promotes systemic mononuclear phagocyte activation and type I IFN production, restricting viral replication in the brain (37). Moreover, the commensal microbiota may regulate viral regionalization along the intestinal tract. The microbiota inhibits the viral infection of MNV in the proximal small intestine, but in the distal regions, including the ileum and colon, it can promote the infection. The regional difference in MNV infection may result from distinct regional expression patterns of the bile acid receptors that regulate type III interferon. The viral inhibition reported in the proximal gut is due to priming a type III IFN response bacteria-biotransformed bile acids (38). Therefore, commensal bacteria may play more complex and protective roles depending on the enteric virus.

On the other hand, another study shows that enteric viruses can restore the beneficial effects of the commensal microbiota in microbiota-depleted mice. MNV infection of germ-free and antibiotics-treated mice can restore intestinal morphology, and lymphocyte function observed in the absence of microbiota without inducing overt inflammation and disease (39). These beneficial effects are attributed to the activation of the IFN-α pathway by MNV, which can also be protective against superinfection with *C. rodentium* and DSS-induced colitis (**Figure 1B**) (39). Thus, the role of some enteric viruses needs to be further explored to elucidate in which context may be beneficial for the host.

## The Role of SCFA in Gut Homeostasis and Infection

As mentioned above, commensals play a key role in generating SCFAs such as butyrate, propionate, and acetate, which can imprint anti-inflammatory and antimicrobial functions in immune cells through the binding with G protein-coupled receptors such as FFAR-2 (also known as GPR43) and FFAR-3 (also known as GPR41), also *via* the modulation of histone deacetylase activity. Some of these anti-inflammatory roles include inducing the differentiation of colonic T_regs_ and peripheral T_regs_, acting as an important negative regulator of autophagy, and exerting immunomodulatory functions in macrophages (40, 41). For instance, butyrate induces macrophage differentiation and changes in their metabolic profile, reducing glycolysis and mTOR activity and increasing antimicrobial responses (42, 43). Administration of antibiotics results in SCFA reduction leading to subsequent hyperresponsiveness of colonic macrophages and increased Th1 responses, which were prevented after butyrate administration (43). In addition, administration of butyrate ameliorates dextran sodium sulfate (DSS)-induced colitis, whereas propionate or an SCFA mix ameliorates colitis in a T cell transfer model of colitis in an FFAR-2-dependent manner (44, 45). Thus, microbiota-derived metabolites are essential in maintaining gut immune responses and preventing gut inflammation.

SCFA has also been linked with inducing antimicrobial functions in different innate cells and inducing protection against various enteric pathogens. Butyrate exerts protective *in vivo* effects against *S.* Typhimurium and *C. rodentium* by inducing colonic macrophage antimicrobial responses (42, 46). Similarly, butyrate-treated macrophages exhibited increased autophagic flux and autophagosome degradation of *S.* Typhimurium together with increased ROS activity (42). Moreover, SCFA also induces antimicrobial functions in type 3 innate lymphoid cells, which play important roles in regulating mucosal immune responses during health and disease by producing IL-22 and IL-17 (47, 48). Acetate has been shown to protect against *C. rodentium* by inducing IL-22 production in ILC3 in an FFAR2-dependent manner (49). In line with this, mice deficient in FFAR2 exhibit defective expansion of ILC1 and ILC3 in the small intestine, colon, and mLN during *C*. rodentium infection, which was not restored by the administration of SCFA, indicating that ILC subsets require SCFA signaling during the response against this bacterium. Importantly, FFAR2^-/-^ mice also exhibit a decreased expansion of splenic ILC1 during *C. rodentium* infection, indicating that defective FFAR2 signaling in ILC may lead to systemic defects during enteric infection (50). In addition, acetate can promote antimicrobial responses against *C. difficile* infection by activating both neutrophils and ILC3 responses in an FFAR-2-dependent manner (51). Taken together, this evidence supports a key role of SCFA in protection against enteric infection by modulating antimicrobial immune responses in specific immune cells such as macrophages, ILCs, and neutrophils.

On the other hand, SCFA has protective effects against enteric pathogens such as *S.* Typhimurium, *C. rodentium*, and *C. difficile* by directly inhibiting their growth. Bacteroides-derived propionate can inhibit *S.* Typhimurium growth, and administration of Bacteroides confers resistance to *S.* Typhimurium infection in a propionate-dependent manner (52). Butyrate-producing microbiota and *in vivo* supplementation with butyrate confers protection against *C. rodentium* infection, which could be mediated by direct effects of butyrate on *C. rodentium* growth (53). In line with this, a diet deficient in dietary fiber increases bacterial species able to degrade intestinal mucins, favoring colonization of *C. rodentium* and lethal colitis (54). Similar beneficial effects of SCFA have been reported in murine models of *C. difficile* infection, as commensals can degrade dietary plant polysaccharides and produce SCFA outcompete *C. difficile* (**Figure 1C**) (55). Another explanation for the beneficial effect of SCFA is the induction of an acidic environment in the intestinal lumen, which inhibits Enterobacteria replication by mediating intracellular acidification (56). Indeed, loss of SCFA leads to an increased pH in the lumen and increased expansion of Enterobacteria in antibiotic-treated mice (56). These findings highlight the important role of SCFA in preventing enteric infection. The following section will explore the importance of microbiota and associated metabolites in preventing lung infection.

## Beneficial Roles of Scfa and Microbiota in Lung Homeostasis and Disease

The gut microbiota plays a protective role against lung infections, particularly in preventing viral infections. Antibiotic-treated mice are more susceptible to the Influenza virus due to the impaired induction of antiviral responses in lung macrophages and subsequent adaptive antiviral responses (57). Furthermore, antibiotic-treated mice exhibit reduced generation of adaptive immune responses following Influenza A virus (IAV) infection, but interestingly, the antibiotic treatment does not affect the generation of CD4^+^ and CD8^+^ T cell responses against the bacterial pathogen *Legionella pneumophila* (58). These effects are attributed to the loss of Toll-like receptor (TLR) stimulation by intestinal Gram-positive bacteria, which are responsible for the activation of the inflammasome and the subsequent production of IL1-β and IL-18 and DC migration from the lung to the lung-draining lymph nodes during IAV infection (58). A similar role has been described for commensals belonging to the *Bacteroidetes* phylum, which induce IFN-β on colonic dendritic cells (DCs) in a TLR-4-TIR-domain-containing-adaptor-inducing IFN-β (TRIF)-dependent manner, subsequently promoting systemic type 1 interferon production and enhancing resistance to IAV infection (59). Of note, although antibiotic treatment affects immune responses during viral infection, if the antibiotic treatment ceases before the infection and is followed by re-colonization, effective immunity against IAV is restored in young mice (60). Taken together, this evidence highlights the important role of the gut microbiota in providing beneficial signals that prevent respiratory infections.

Recent studies have shown beneficial effects of metabolites in lung infection by modulating immune cell responses. For example, it has been demonstrated that the microbial metabolite desaminotyrosine is protective against influenza infection by inducing type I IFN signaling and that administration of desaminotyrosine-producing bacteria restores immunity against Influenza in antibiotic-treated mice (61). Importantly, the bacteria used in this study was the human associated gut bacteria *Clostridium orbiscindens*, which can degrade flavonoids, suggesting that the human microbiota could modulate influenza infection. In line with this, antibiotic treatment has been linked with impaired immunity to H1N1 virus immunization in a clinical trial in healthy humans treated with a broad spectrum cocktail of antibiotics prior to influenza vaccination, which was associated with a reduction in circulating metabolites, including a drastic decrease of secondary bile acid, and increased inflammasome activation (62). In addition, subjects with low pre-existing antibody titers against influenza and treated with antibiotics exhibited impaired IgA and IgG responses against influenza following vaccination (62). Therefore, this evidence indicates that the human commensal bacteria and derived metabolites are likely candidates for the regulation of systemic immunity against influenza infection.

SCFA are present at high concentrations in the gut, mainly in the caecum and colon, ranging from 80-130mmol/kg in humans whereas blood concentrations are low (63). SCFA measurement in human and mouse lung sections suggests that acetate and propionate are the most abundant SCFA in the lung although with variable concentrations between 10-50mM for acetate and 1-10mM for propionate (64). Germ-free mice lack lung SCFA, which is restored after monocolonization with the gram-negative gut bacteria *Bacteroides thetaiotaomicron*, which indicates that SCFA are produced by the gut microbiota and then circulate through the blood to the lung (64). Furthermore, SCFA can directly modulate immune cell responses in the lung. A high dose of propionate (5mM) reprograms the metabolism of LPS-exposed alveolar macrophages, decreasing their metabolic stress but a lower concentration (0.5mM) can further increase their metabolic stress, associated with increased glycolysis (64). In line with this, high concentrations of propionate prevent inflammatory cytokine secretion in primary airway epithelial cells but low concentrations may indeed induce their secretion (65). Additionally, high concentrations of SCFA (50-100mM) may impair the growth of the opportunistic pathogen *Pseudomonas aeruginosa in vitro* but low concentrations may indeed favor their growth (65). Low concentrations of SCFA (around 2mM) have been reported in the sputum of patients with cystic fibrosis, which suggest that low concentrations of SCFA may contribute to the airway alterations observed in this disease (65). Thus, the gut microbiota may play an important role as a source of systemic SCFA and provide sufficient concentrations to modulate lung immune responses during health and infection.

In line with the role of the gut microbiota modulating lung infection, high fiber diet and administration of SCFA also modulate lung infection in mouse models. A high fiber diet and acetate administration generated protection against allergic airway disease in a house-dust mite (HDM)-induced allergy model, which prevented inflammation mediated by eosinophils. The acetate receptor FFAR-3 expressed on dendritic cells (DC) mediated this protective effect and prevented their exacerbated activation (66). Additionally, a high fiber diet enhanced the generation of Ly6C^–^patrolling monocytes, which differentiate into alternatively activated macrophages and reduce neutrophil recruitment into the lung during the influenza infection (66). Similarly, acetate administration induced interferon-β and subsequent antiviral responses in the lung through FFAR-2 and IFNAR signaling pathways, which resulted in protection against respiratory syncytial virus (RSV) in mice (67). Acetate also induces protection in mice against human clinical isolates of RSV, which was dependent on the expression of the viral sensor retinoic-acid inducible gene I (RIG-I) (68). Furthermore, pre-treatment of human pulmonary cells and nasopharyngeal aspirate cells with acetate is protective against infection with the same RSV isolates increasing the expression of the RIG-I and interferon-stimulated genes (68). In the context of bacterial pneumonia, the gut microbiota and derived metabolites may play protective effects as depletion of the gut microbiota causes increased susceptibility to *Streptococcus pneumoniae* infection, and alveolar macrophages exhibit diminished phagocytic activity (69). Furthermore, fecal microbiota transplantation restored normal immune responses against pneumococcal infection. This is consistent with data showing that acetate administration is protective during *Klebsiella pneumoniae* infection in an FFAR-2-dependent manner (70). Taken together, this evidence highlights the important role of the intestinal microbiota and derived metabolites in lung resistance to viral infection.

## Local and Distal Effects of Enteric Infection

Pathogen-driven intestinal inflammation can induce drastic local changes in the diversity and abundance of intestinal microbiota. For example, infection with *C. rodentium* reduces the total intestinal microbiota and alters the diversity of bacterial phyla at the peak of the infection (7 days), increasing the abundance of Enterobacterales and reducing the abundance of Bacteroidales (71). Similarly, *S.* Typhimurium also induces an increase in the abundance of Enterobacteria together with changes in caecal metabolites in murine models containing murine or humanized microbiota (15, 72–74). However, no changes were observed in mice infected with *Campylobacter jejuni*, which cannot induce intestinal inflammatory responses (71). In both infection models, it has been reported that changes in the intestinal microbiota are not permanent, and restoration of normal microbiota abundance is observed after the peak of the infection (71, 73). Interestingly, similar effects were observed in a model of chemically induced colitis using dextran-sodium sulfate (DSS) and also in IL-10 deficient mice (IL10-/-), both exhibiting increased susceptibility to non-pathogenic *E. coli* (71). Furthermore, DSS-induced colitis also favors infection with *C. rodentium* (14). Thus, both pathogen-driven and chemically induced intestinal inflammation alter the microbiota composition and may further impact susceptibility to enteric pathogens.

Recent studies in patients colonized or infected with *C. difficile* have shown that the infection affects the gut microbiota and metabolome. *Veillonella* is increased in patients colonized and infected with *C. difficile*, whereas *Eubacterium hallii* and *Fusicatenibacter* are more abundant in control patients without *C. difficile* colonization at hospital admission, suggesting that these bacteria may confer resistance to the colonization and infection (75). Infected patients exhibit a distinctive fecal metabolome, with increased abundance of the SCFA 4-methylpentanoic acid, a product of leucine fermentation, and reduced abundance of the secondary bile acids (76) which may reflect either the lack of beneficial commensals involved in the production of these bile acids and/or that they may confer resistance to infection by inhibiting *C. difficile* germination and growth, as observed in mice (30, 31). Thus, these changes in microbiota and susceptibility to non-pathogenic bacteria could be attributed to the host intestinal inflammation and could lead to alterations in the intestinal immune system and cause changes in immune responses in other tissues.

The distal consequences of enteric infection are still incompletely understood. Few studies have analyzed changes in immune responses in distal tissues such as the lung, but some of them have linked *S.* Typhimurium with protection from allergic lung inflammation in mice (77). This effect seems to be mediated by the increase in a heterogeneous population of CD11b^+^ Gr1^+^ myeloid cells, which expand after *S*. Typhimurium infection and reduce Th2 responses (78). However, previous studies have not reported that other common models of enteric bacteria such as *C*. *rodentium* exhibit changes in lung immunity and/or dysbiosis. Similarly, very little is known about the distal effects of enteric viruses. A recent study compared the impact of different enteric viruses in germ-free mice and showed changes in the abundance of lung immune cells following enteric infection. In particular, mice infected with murine adenovirus (MAdV1) or reovirus (TL1) exhibit a reduction of lung naive T cells accompanied by an increase in effector memory CD4^+^ and CD8^+^ T cells, whereas mice infected with astrovirus or parvovirus exhibit increased frequency of cDCs and macrophages but a decrease in B cells (79). Further research is required to understand whether these changes affect the host susceptibility to secondary lung infection.

The role of intestinal pathobionts is a matter of interest due to their complex roles regulating both regulatory and inflammatory responses and may also generate distal effects, including immune cell migration between both tissues. Members of the phylum Proteobacteria (including *E. coli*) regulate the production of the alarmins IL-33 and IL-25 and, in turn, modulate the migration of ILC2, which are important mediators of type 2 immune responses by the secretion of IL-4, IL-5, and IL-13 (80). Mice treated with a mixture of antibiotics (streptomycin, colistin, ampicillin, and vancomycin) exhibited increased frequencies of small intestinal ILC2s and reduced frequencies of lung ILC2. In contrast, treatment with ampicillin or models of sepsis increased the abundance of Proteobacteria in the small intestine resulting in increased production of IL-33 and IL-25 and elevated frequencies of lung ILC2. Furthermore, IL-33 induces increased migration of a tissue-resident ILC2 population (expressing the IL-33 receptor ST2) from the small intestine to the lung in response to the chemokine CXCL16, which binds the homing receptor CXCR6, whereas IL-25 induces the migration of inflammatory ILC2 (ST2^-^ KLRG1^+^) into the gut in response to the chemokine CXCL25 and the homing receptor CCR9. Importantly, blocking CCR9 or the IL-33 receptor ST2 (expressed on ILC2) is detrimental during sepsis, causing decreased frequencies of ILC2 in both lung and small intestine and resulting in more severe tissue damage, indicating that ILC2 migration plays a protective role. In line with this, infection with the intestinal helminth *Trichinella spiralis* induces inflammatory ILC2 in an IL-25-dependent manner in the small intestine, which in turn migrate to the lung and subsequently drive increased production of the mucins Muc5ab and Muc5b in an IL-13-dependent manner (81). This increased mucus production is protective against secondary infection with the lung helminth *Nippostrongylus brasiliensis*, limiting the parasite migration. These findings indicate that the microbiota and/or infectious diseases may drive ILC2 migration between the lung and the small intestine, which role may be beneficial in limiting inflammation and secondary infection.

Other members of the phylum Proteobacteria have been related to immunomodulatory properties in the intestine, which may modulate lung immune responses. Members of the family *Helicobacter*, colonizing the gastrointestinal tract, play a complex role in the induction and modulation of distal inflammation. *H. hepaticus* has been reported as a colonizer of the lower bowel in immunocompetent mice, driving IL-23-dependent colitis in mouse models defective in IL-10 signaling (82). This pathobiont can induce changes in the gut microbiota and generate a higher frequency of neutrophils in the spleen and lung (83). Mice naturally colonized with *H. hepaticus* exhibited increased susceptibility to *Mycobacterium tuberculosis* infection, accompanied by increased CD4^+^ T cell infiltration in the lungs (83). Interestingly, recent evidence supports the role of *Helicobacter hepaticus* and *Helicobacter typhlonius* in inducing tolerogenic responses in macrophages and T cells, suggesting they may play a dual role depending on the context (84–86). Furthermore, pathobionts such as members of the genera *Helicobacter* and *Deferribacter* could be related to maintaining intestinal and circulating metabolites (87, 88). Thus, these species can play complex roles in either protection or susceptibility to other infectious diseases in distal tissues such as the lung.

Moreover, murine models of chemically induced acute colitis display lung inflammation due to changes in circulating inflammatory cells and alterations in gut permeability. Experiments using DSS and TNSB as colitis inducers increased the egress of neutrophils from the bone marrow and subsequent recruitment into the lung, mediated by increased secretion of IL-6 (89). In addition, similar experiments using DSS colitis and anti-CD40-induced colitis have shown that expression of platelet-activating factor receptor (PAFR) is increased in lung neutrophils and epithelial cells, resulting in lung neutrophilia and increased NLRP3 inflammasome activation and production of IL-1β. These changes seem to be mediated by bacterial phosphorylcholine, which can bind and activate PAFR, as treatment with antibiotics during DSS colitis reduced inflammasome activation in lung cells (90). These results suggest that after DSS colitis, there may be increased bacterial translocation to the lung, inducing lung inflammation. Altered gut permeability has also been reported in models of stroke in aged mice, which exhibit increased bacterial translocation into the lung (91). Thus, if an enteric pathogen generates extensive damage and increases gut permeability, it is likely that bacterial commensals and/or pathobionts may enter into the circulation and infect distal tissues. Further research is required to confirm this hypothesis.

## Distal Effects of Viral Lung Infection

The link between the intestine and lung is increasingly appreciated, and several studies have addressed the impact of lung infection in both the gut microbiota and intestinal immune responses. Several studies have described changes in the microbiota during influenza infection in mouse models and humans. Influenza A drastically affects intestinal microbiota abundance, increases antimicrobial peptides’ production in Paneth cells, and reduces mucus layer integrity (92). In addition, these changes in the gut microbiota are accompanied by a reduction of cecal and circulating SCFA 7 days post-infection, but normal levels are restored after 14 days (93). Similarly, the gut microbiome analysis in patients with H1N1 and H7N9 Influenza through fecal samples has shown that microbial diversity is decreased together with an increase in the relative abundance of opportunistic pathogens compared to healthy individuals (94, 95). In H1N1 Influenza, increase the abundance of opportunistic pathogens such as *Escherichia*, *Shigella*, *Finegoldia*, *Anaerococcus*, and *Prevotella*, and reduce the relative abundance of beneficial symbionts has been reported (94). In line with this, another study showed alterations in the gut microbiota of patients with H7N9 Influenza, who showed an increase in potentially pathogenic bacteria such as *Escherichia coli*, *Salmonella*, *Enterococcus*, and *Veillonella* together with a decrease in *Ruminococcus*, *Eubacterium*, *Roseburia*, and *Bifidobacterium.* Even further alterations in the gut microbiota were observed in infected patients treated with antibiotics (95). Alterations in the intestinal microbiota during influenza infection, particularly the increase in Enterobacterales, results in exacerbated inflammatory Th17 responses in the small intestine and induction of intestinal injury. This is caused by increased migration of lung-derived T cells into the gut, which migrates in a CCR9-dependent manner (96). Thus, it has been hypothesized that lung infection may increase the gut homing of inflammatory cells into the gut. Moreover, mice infected with IAV also exhibit increased gut permeability and increased blood levels of lipopolysaccharide-binding protein (LBP), an acute-phase protein produced in the liver that induces immune responses (97). In addition, decreased levels of SCFA were observed 7 days post-infection and administration of SCFA partially reverted altered intestinal permeability in IAV-infected mice (97). Therefore, influenza infection may affect immune cell migration and gut permeability, which in turn facilitates bacterial translocation into the blood.

**Figure 2:**
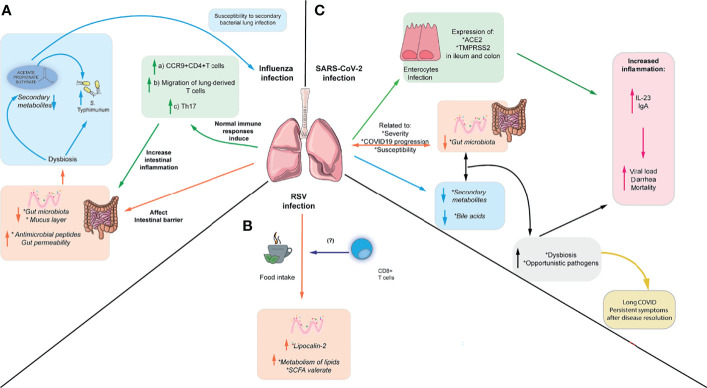
Respiratory viruses affect intestinal immunity and intestinal microbiota. **(A)** IAV can cause intestinal inflammation through the migration of inflammatory Th17 cells to the gut, which in turn cause microbiota-dependent inflammation. In addition, IAV can cause increased susceptibility to *S.* Typhimurium infection and increased susceptibility to *S. pneumoniae*, which can be reversed by administering SCFA. **(B)** RSV can cause changes in enteric antimicrobial peptides such as lipocalin-2 and low-grade intestinal inflammation. Also, it can alter the microbiota and metabolites through the changes in food intake during infection. These changes in food intake have been linked to alterations in CD8^+^ T cells **(C)** SARS-CoV-2 can alter the microbiota composition, increasing the abundance of opportunistic pathogens and altering the metabolome. Furthermore, it can directly infect intestinal epithelial cells and affect levels of fecal cytokines, although it is unclear if it can cause intestinal inflammation. Moreover, gut dysbiosis and bacterial translocation of opportunistic gut pathogens into the blood have been associated persistent symptoms after disease resolution.

Furthermore, microbiota alterations observed in IAV infection led to increased susceptibility to a murine model of *S.* Typhimurium superinfection (92). A similar study showed weight loss and increased mortality following secondary infection with *S.* Typhimurium and supplementation with SCFA limited bacterial dissemination and improved mouse survival but did not reduce cecal bacterial load. These findings suggest that SCFA do not affect S. Typhimurium growth in this context but can ameliorate its systemic effects (97). In addition, susceptibility to a secondary infection with *S.* Typhimurium following influenza infection has been linked to the induction of antiviral IFN-I, which promotes gut dysbiosis (98). Moreover, changes in the microbiota and SCFA observed in IAV increased mice susceptibility to secondary respiratory bacterial infection. Mice treated with antibiotics and then recolonized with microbiota from Influenza A-infected mice exhibit higher susceptibility to a secondary infection with *S. pneumoniae*, attributed to the altered bactericidal activity of alveolar macrophages. Supplementation with acetate is protective against this secondary bacterial infection and a co-infection with both IAV and *S. pneumoniae* in an FFAR-2-dependent manner (**Figure 2A**) (93). Therefore, changes in lung immune responses during respiratory infection may directly or indirectly affect gut microbiota and gut immunity.

Moreover, although hRSV does not infect the gut, the intestinal microbiota is altered during infection, and the colon exhibits a low grade of inflammation in mice. hRSV infection induces higher expression of lipocalin-2 in the intestine, which is an important antimicrobial peptide mediating iron sequestration and inducing iron starvation in bacteria (99). However, these changes do not render increased susceptibility to the enteric pathogen *C. rodentium* (100). Furthermore, hRSV infection also induces changes in the fecal metabolites associated with lipid metabolism, including enrichment in sphingosines, sphingomyelins, ceramides, polyunsaturated fatty acids, the SCFA valerate, and reduction in primary and secondary bile acids. Interestingly, these changes in gut microbiota and metabolites seem to be induced by the reduced food intake during hRSV infection but increased viral load does not correlate with weight loss, suggesting that inappetence is driving the reduced food intake. Depleting CD8^+^ T cells reduced weight loss during the infection and prevented changes in the gut microbiota, suggesting that CD8^+^ T cells play a role in inappetence during viral infection. However, it is unclear which cytokine may be responsible for this effect (**Figure 2B**) (100). This is consistent with data indicating that a diet-imposed weight loss recapitulates in part the changes in gut microbiota and reduction in SCFA observed during IAV infection (93), supporting the idea that reduced food consumption during viral infection may alter the gut microbiota and derived metabolites. Moreover, alterations in the gut microbiota correlate with disease severity in children with RSV bronchiolitis whereas patients with milder symptoms exhibit higher levels of acetate in the stool (68, 101), suggesting that the composition of the microbiota and SCFA may confer protection against RSV infection in humans. It is still unclear whether these changes in the human microbiota during RSV infection are due to the inappetence reported in mice or other alternative mechanisms.

Emerging evidence has shown the importance of gut microbiota and its metabolites in the severity of COVID-19 disease in animal models and humans. Different animal models able to express the SARS-CoV-2 receptor angiotensin-converting receptor 2 (ACE2) including macaques, hamsters and transgenic K18-hACE2 mice have been used to recapitulate human COVID-19, explore changes in the gut microbiota and complement data from clinical human studies. In macaques infected with SARS-CoV-2, the alterations in the gut microbiota peaked at day 13 post-infection, with an increased relative abundance of the genus *Acinetobacter* (from the phylum Proteobacteria) and the family Ruminococcaceae (from the phylum Firmicutes), both correlating with the presence of SARS-CoV-2 in the upper respiratory tract (102). Besides, targeted quantitative metabolomics revealed a drop in SCFAs and changes in several bile acids and tryptophan metabolites. These variations in fecal SCFAs concentration might reflect differences in their use by host cells and/or/or production by gut bacteria. Relative abundance of bacteria from the Ruminococcaceae family and other taxa known to be SCFA producers negatively correlated with systemic inflammatory markers, suggesting that SCFAs could be protective against a SARS-CoV-2 infection (102). A model of SARS-CoV-2 infection in hamsters also exhibits gut dysbiosis characterized by a reduction in SCFA producers from the families Ruminococcaceae and Lachnospiraceae and a systemic drop in SCFAs. However, supplementation with SCFA failed to improve disease outcome in this model (103). K-18-hACE2 transgenic mice also display alterations in the cecal microbiota, with a decrease in the families Lachnospiraceae and Oscillospiraceae and an increase in the family Akkermansiaceae, although is unclear whether these mice also exhibit changes in SCFA and other metabolites (104). Taken together, this evidence supports the idea that SARS-Cov-2 causes alterations in the gut microbiota of different animal models.

Several studies have analyzed the gut microbiota of COVID-19 patients. Gut microbiota associated with blood proteomic biomarkers has been identified in patients with COVID-19 (severe and non-severe), in order to predict COVID-19 progression to clinically severe phase, which correlated with inflammatory biomarkers such as IL-6, IL-1β, TNF-α, and high-sensitivity C-reactive protein (hsCRP) (105). Members of the gut microbiota positively associated with inflammatory biomarkers include the genus *Ruminococcus*, *Blautia*, and *Lactobacillus*, whereas the genus *Bacteroides*, *Streptococcus*, and the order Clostridiales were negatively associated with disease severity (105). This gut microbiota was closely related to fecal metabolites, in particular with those associated with amino acid metabolism, which in turn might be related to the systemic proteomic profile associated with COVID-19 severity (105). Similarly, analysis of the fecal microbiome of COVID-19 patients who were hospitalized showed that pathogens and opportunistic pathogens were enriched in the gut microbiome, including *Hungatella hathewayi*, *Bacteroides nordii*, and *Actinomyces viscosus*. *Clostridium ramosum* and *H. hathewayi* were the most abundant bacteria positively associated with COVID-19 disease severity, associated with human infection and bacteremia (106). Also, members of Firmicutes phylum have been associated with disease severity (106). In contrast, two beneficial species, *Alistipes onderdonkii* and *Faecalibacterium prausnitzii*, were the most abundant bacterial species that negatively correlated with COVID-19 severity (106). Furthermore, another study analyzing the fecal microbiota of patients with COVID-19 reported a reduction of beneficial symbionts together with an increase in opportunistic bacteria such as *Escherichia*, *Shigella*, *Streptococcus*, *Rothia*, *Veillonella*, and Actinomycetes (94, 107). Thus, patients with COVID-19 exhibit gut dysbiosis and alteration in fecal metabolites, although further research is required to understand how SARS-CoV-2 alters the composition of the gut microbiota.

Several works have postulated that gut dysbiosis may lead to bacterial translocation into the blood and contribute to COVID-19 severity. K18-hACE2 mice infected with a high dose SARS-CoV-2 also exhibit alterations in gut permeability and defective Paneth cell function associated an increase in Akkermansiaceae (108). Similarly, blood culture analysis of COVID-19 patients revealed bloodstream infection, which matched with bacterial species found in the stool from the same patients, supporting the idea that gut bacteria can translocate into the blood during SARS-CoV-2 infection. In addition, *Faecalibacterium* was negatively associated with bloodstream infection, suggesting that their decrease during disease may favor bacterial translocation (108). Furthermore, increased levels of markers of bacterial and fungal translocation together with increased levels of markers of tight junction permeability have been reported in patients with severe COVID-19, indicating that altered gut permeability may allow bacterial translocation into the blood and contribute to disease severity (109). This potential mechanism may contribute to the persistency of COVID-19 symptoms in patients who had severe COVID-19 following disease resolution. Patients exhibiting respiratory dysfunction 3 months after severe COVID-19 exhibit gut dysbiosis including an increase in *Veillonella*, which could be related to pulmonary fibrosis. In addition, they display high levels of LBP in blood, which could be indicative of altered gut permeability (110). Moreover, patients with post-acute COVID-19 syndrome (PACS) exhibit persistent symptoms up to 6 months and it has been recently shown that their microbiota profile at admission can be predictive of PACS. These patients exhibit gut dysbiosis 6 months after disease resolution and persistent respiratory symptoms correlated with the presence of the opportunistic gut pathogens *Streptococcus anginosus, Streptococcus vestibularis, Streptococcus gordonii and Clostridium disporicum* (111). In contrast, patients without PACS exhibited an enrichment in species from the genera *Bifidobacterium*, *Blautia* and *Bacteroides* and negatively correlated with PACS, indicating that the composition of the microbiota may affect susceptibility to long-term complications of COVID-19 (111) (**Figure 2C**). On the other hand, patients with severe COVID-19 exhibited reduced abundance of *F. prausnitzii* together with a reduction in the biosynthesis of SCFAs and the aminoacid L-isoleucine, which persisted 30 days after disease resolution (112). Butyrate and L-isoleucine exhibited a negative correlation with disease severity and pro-inflammatory markers, suggesting that reduction in beneficial commensals able to secrete these metabolites may contribute to systemic inflammation and persistent symptoms (112). Further research is required to define the mechanisms by which the gut microbiota and associated metabolites can prevent or increase susceptibility to SARS-CoV-2 infection.

Patients with SARS-CoV-2 infection exhibit gastrointestinal symptoms, and several studies suggest the presence of this virus in feces and potential fecal-oral transmission (94, 113). Studies in the Chinese population have reported gastrointestinal symptoms in up to 50% of COVID-19 infected cases (114). Indeed, the virus can infect human enterocytes *in vitro*, as they express ACE-2, targeted by the viral glycoprotein spike (S), which can trigger up-regulation antiviral responses (115). In addition, immunofluorescence studies have detected higher expression of ACE2 in the human ileum compared to the colon. Conversely, the human colon expresses more elevated transmembrane serine protease 2 (TMPRSS2) levels, which cleaves the S protein and could facilitate viral entry into the host cell (116, 117). Expression of both proteins is increased in the colon of patients with IBD, and transcriptomic analysis in these patients indicates that both are involved in metabolic and immune functions, including type 1 interferon signaling (116). In line with this, patients with SARS-CoV-2 display high fecal IL-8 and low levels of IL-10, although they do not correlate with more severe gastrointestinal symptoms. In addition, detection of higher viral load in feces has been associated with diarrhea and mortality in COVID19 patients, and severe patients exhibit high levels of the pro-inflammatory cytokine IL-23 in feces and increased anti-SARS-CoV-2 IgA, which could reflect either a systemic response to the virus or localized intestinal immune responses (**Figure 2C**) (116). Hamsters infected with SARS-CoV-2 also display increased expression of anti-microbial peptides, IFN-γ and IFN-stimulated genes in the colon but no intestinal damage was found by histology, suggesting that SARS-CoV-2 induces mild intestinal inflammation (103). Taken together, these findings suggest that the virus may be able to directly infect intestinal epithelial cells and may lead to gastrointestinal symptoms.

Moreover, bacterial respiratory infections also impact the intestinal microbiota. Studies in murine models of tuberculosis (TB) have reported decreased gut microbiota diversity in mice infected with TB (118, 119). Pediatric patients with tuberculosis exhibit a reduction of beneficial commensal microorganisms and an increased abundance of enteric pathogens such as *Prevotella* and *Enterococcus* (120). A study in adult patients also showed alterations in the microbiota with reduced Bacteroidetes and increased Actinobacteria and Proteobacteria in patients with recurrent tuberculosis. In the same study, both new and recurrent tuberculosis cases exhibited a decreased abundance of *Prevotella* and *Lachnospira* (121). In addition, African patients infected with *Mycobacterium Africanum* exhibit distinct gut microbiota compared to those infected with *Mycobacterium tuberculosis*, with an increased abundance of Enterobacterales (122). Of note, it must be considered that studies in human patients may also reflect changes that could be attributed to the tuberculosis treatment, which may also affect the intestinal microbiota (119, 122). None of the studies mentioned above have addressed changes in intestinal immune cells, and it is unclear whether bacterial respiratory infections can affect intestinal immunity.

## Conclusion

Infectious diseases affecting mucosal barriers such as the intestine and the lung may have distal effects due to the pathogen’s direct or indirect impact. In the case of lung respiratory infections, several viruses have been described to impact either the intestinal microbiota or intestine immunity through direct and indirect mechanisms. Influenza infection may affect the migration of inflammatory lung T cells into the gut, provoking exacerbated Th17 responses and intestinal injury in a microbiota-dependent manner. In addition, influenza increases gut permeability in mouse models of infection, facilitating bacterial translocation into the blood. In contrast, SARS-CoV-2 seems to directly infect intestinal epithelial cells, which could be responsible for the gastrointestinal symptoms reported in COVID-19 patients. All these viruses and some bacterial respiratory pathogens impact the composition of the gut microbiota and associated metabolites, which could be explained by either the reduced food intake, as reported in RSV and influenza infection, and/or indirect effects of the respiratory pathogen, as observed in the IFN-I-induced dysbiosis reported in influenza infection. These alterations in the gut microbiota may have long term effects as observed in cases of severe and persistent COVID-19, which display increased presence of opportunistic gut pathogens. These bacteria can translocate into the blood due to altered gut permeability and lead to persistent respiratory symptoms following disease resolution. It is unclear whether these bacteria translocate into the lung or whether their effect on persisting symptoms may be related to the increased bacterial-derived factors in circulation and subsequent increased levels of systemic inflammatory signals.

Moreover, the effects of enteric infections in lung immunity are still poorly understood. Although extensive evidence links changes in the intestinal microbiota and derived metabolites with systemic protective effects against respiratory infections, few works have linked common models of intestinal inflammation such as *C. rodentium* with altered lung immunity. *Salmonella* has been linked with protective effects against allergy due to the protective effect of CD11b^+^ Gr1^+^ myeloid cells. Similarly, migration of ILC2 between the lung and the gut seems to be coordinated by species of the Proteobacteria phylum and may be protective during sepsis and secondary helminth infection. In contrast, members of the Helicobacter genus exhibit immunoregulatory properties, which may render increased susceptibility to bacterial pneumonia. Furthermore, acute intestinal inflammation may drive changes in gut permeability, facilitating bacterial translocation into the lung. Further research is required to understand how intestinal and lung infectious diseases alter the local and systemic metabolome and how it may impact distal mucosal barriers.

## Author Contributions

All authors listed have made a substantial, direct and intellectual contribution to the work, and approved it for publication.

## Funding

This study was supported by the Millennium Institute on Immunology and Immunotherapy (P09/016-F; ICN09_016) and by Agencia Nacional de Investigación y Desarrollo de Chile (ANID) through Fondo Nacional de Desarrollo Científico y Tecnológico (FONDECYT) grant n° 1190830 (to AK) and n° 11200764 (to FM-G)

## Conflict of Interest

The authors declare that the research was conducted in the absence of any commercial or financial relationships that could be construed as a potential conflict of interest.

The editor BP declared a past co-authorship with the author SB.

## Publisher’s Note

All claims expressed in this article are solely those of the authors and do not necessarily represent those of their affiliated organizations, or those of the publisher, the editors and the reviewers. Any product that may be evaluated in this article, or claim that may be made by its manufacturer, is not guaranteed or endorsed by the publisher.
